# Transportability and Implementation Challenges of Early Warning Scores for Septic Shock in the ICU: A Perspective on the TREWScore

**DOI:** 10.3389/fmed.2021.793815

**Published:** 2022-02-08

**Authors:** Michael S. A. Niemantsverdriet, Meri R. J. Varkila, Jacqueline L. P. Vromen-Wijsman, Imo E. Hoefer, Domenico Bellomo, Martin H. van Vliet, Wouter W. van Solinge, Olaf L. Cremer, Saskia Haitjema

**Affiliations:** ^1^Central Diagnostic Laboratory, University Medical Center Utrecht, Utrecht University, Utrecht, Netherlands; ^2^SkylineDx, Rotterdam, Netherlands; ^3^Department of Intensive Care Medicine, University Medical Center Utrecht, Utrecht University, Utrecht, Netherlands

**Keywords:** early warning score (EWS), TREWScore, sepsis, septic shock, intensive care

## Abstract

The increased use of electronic health records (EHRs) has improved the availability of routine care data for medical research. Combined with machine learning techniques this has spurred the development of early warning scores (EWSs) in hospitals worldwide. EWSs are commonly used in the hospital where they have been developed, yet few have been transported to external settings and/or internationally. In this perspective, we describe our experiences in implementing the TREWScore, a septic shock EWS, and the transportability challenges regarding domain, predictors, and clinical outcome we faced. We used data of 53,330 ICU stays from Medical Information Mart for Intensive Care-III (MIMIC-III) and 18,013 ICU stays from the University Medical Center (UMC) Utrecht, including 17,023 (31.9%) and 2,557 (14.2%) cases of sepsis, respectively. The MIMIC-III and UMC populations differed significantly regarding the length of stay (6.9 vs. 9.0 days) and hospital mortality (11.6% vs. 13.6%). We mapped all 54 TREWScore predictors to the UMC database: 31 were readily available, seven required unit conversion, 14 had to be engineered, one predictor required text mining, and one predictor could not be mapped. Lastly, we classified sepsis cases for septic shock using the sepsis-2 criteria. Septic shock populations (UMC 31.3% and MIMIC-III 23.3%) and time to shock events showed significant differences between the two cohorts. In conclusion, we identified challenges to transportability and implementation regarding domain, predictors, and clinical outcome when transporting EWS between hospitals across two continents. These challenges need to be systematically addressed to improve model transportability between centers and unlock the potential clinical utility of EWS.

## Introduction

Early recognition and diagnosis of hospitalized patients at risk of clinical deterioration is crucial for adequate treatment. To aid healthcare professionals in their systematic assessment of these patients, a plethora of flowcharts and early warning scores (EWSs) have been developed for various diseases. Such scores are especially relevant for early detection of potentially life-threatening syndromes where time is of essence (1–3). For sepsis, in particular, several EWS have been developed for the identification of preseptic patients in hospital wards, emergency departments (EDs), and intensive care units (ICUs) (4). The continuous registration and collection of clinical and vital parameters in the ICU provide a unique opportunity to continuously calculate EWS for sepsis and adjust treatment thereupon (5). Consequently, many such scores have been developed and published, albeit almost exclusively in single centers. As EWS are currently not transported and implemented to other centers, the universal potential of these models cannot be fully exploited yet.

Transporting scores between healthcare settings faces several challenges. First, the EWS needs to be openly accessible to reproduce the model. Code is often not publicly shared, including the predictors of EWS and, therefore, either needs to be requested or reverse-engineered (5). Second, no hospital is alike as patient populations tend to be rather hospital-specific based on the size of the hospital, location, and case-mix (4). Moreover, EWS input parameters are not always readily available throughout different hospitals as they are either recorded differently or may not be available at all. Finally, EWS have to be carefully tested to assess their clinical validity in the recipient center, a step that is frequently omitted (4). Broad, international, and interhospital application of algorithm-based EWS will have to tackle and overcome these hurdles.

Even though these challenges are widely recognized in literature, we were interested in addressing and evaluating the scope of these challenges related to the domain, predictor, and clinical outcome (6, 7). As a use case, we attempted to transport the Targeted Real-time Early Warning Score (TREWScore) algorithm proposed by Henry et al. in 2015 to our center (8). The TREWScore was trained on electronic health record (EHR) data from the publicly available Medical Information Mart for Intensive Care (MIMIC)-II database to prospectively identify patients with septic shock in the ICU (9). With a high accuracy [0.83 area under the curve (AUC)] at a median diagnostic lead time of 28.2 h before shock onset, the TREWScore was received with great enthusiasm.

We explored transportability and implementation challenges relating to domain, predictors, and clinical outcomes. This study was performed according to the Declaration of Helsinki, the GDPR and the institutional review board approved of the study (registration number 19/543). Only pseudonymized data were used.

### Domain Challenge

First, we evaluated the domain by comparing the ICU populations of the MIMIC-III and the University Medical Center (UMC ICU) Utrecht. We included 55,330 and 18,013 consecutive ICU stays for MIMIC-III and UMC ICU, respectively. The MIMIC-III is a publicly available database comprising data from the ICU units of the Beth Israel Deaconess Medical Center collected between 2001 and 2012 (10). UMC Utrecht is a large tertiary referral hospital located in Utrecht, Netherlands. From the UMC, ICU included all patients between 2011 and 2019. From both databases, we included consecutive patients older than 18 years, and data were combined for patients who were readmitted to the ICU within 24 h.

The UMC ICU cohort was younger (64.1 vs. 65.8 years) with a higher proportion of men (56.6 vs. 53.3%) (Table 1). ICU length of stay was shorter in the UMC ICU cohort (1.0 vs. 2.2 days), whereas total hospital length of stay was longer in the UMC cohort (9.0 vs. 6.91). Proportions of hospital mortality, blood pressure monitoring, and mechanical ventilation were all higher in the UMC ICU cohort, whereas MIMIC-III had a two-fold higher sepsis prevalence compared to the UMC ICU, 31.8 and 14.2%, respectively. There were thus significant differences in cohort characteristics between MIMIC-III and UMC ICU, with the latter appearing more severely ill.

**Table 1:** Characteristics of intensive care unit stays.

	**MIMIC-III (*N* = 53,330)**	**Of which septic shock (*N* = 4,631)**	**UMC ICU (*N* = 18,013)**	**Of which septic shock (*N* = 794)**
Included years of ICU admission	01-01-2001 / 31-12-2012		01-01-2011 / 30-06-2019	
Distinct patients, count	38,511		17,038	
Hospital admissions, count	49,694		17,195	
Patient characteristics				
Age, years, median [Q1–Q3]	65.8 [52.9–77.9]	67.4 [55.4–79.3]	64.1 [53.0–72.5]	62.0 [52.0–69.0]
Gender, male ICU stays (%)	21,796 (56.6%)	2,469 (53.3%)	11,522 (64.0%)	502 (63.2%)
ICU admissions with at least one sepsis episode during ICU stay, count	17,032 (31.8%)		2,557 (14.2%)	
**Characteristics and outcomes of ICU stay**				
ICU length of stay, median days [Q1–Q3]	2.2 [1.2–4.2]		1.0 [0.8–3.2]	
Hospital length of stay, median days [Q1–Q3]	6.91 [4.0–11.9]		9.0 [5.8–18.2]	
ICU mortality (%)	4,560 (8.6%)		1,623 (9.0%)	
Hospital mortality (%)	5,739 (11.6%)		2,450 (13.6%)	
Invasive arterial blood pressure monitoring, count (%)	39,149 (73.4%)		17,457 (96.9%)	
Mechanical ventilation, count (%)	25,740 (48.3%)		15,549 (86.3%)	
Length of stay of ICU admissions with at least one septic shock period during ICU stay, count Mean		151.3 [68.9–319.8] 244.2		248.8 [97.1–522.7] 423.4
Time to first shock event, median hours [Q1–Q3] Mean		19.6 [8.7–45.7] 53.2		44.4 [21.6–136.3] 138.7

*ICU, Intensive Care Unit*.

### Predictor Challenge

From a total of 54 predictors, the TREWScore automatically selected 26 by removing uninformative predictors with lasso regularization (11). We mapped all 54 TREWScore predictors used for training to our database. As clinical practice differs among centers, not all predictors were recorded for the UMC ICU cohort and/or were measured in a different unit of measure. Apart from missingness and unit discrepancies, TREWScore also comprises engineered predictors based on a combination of predictors and/or International Classification of Diseases (ICD)-9 codes. As ICD-9 codes are not available in the stored routine care data in the UMC ICU cohort, we extracted these predictors from the Dutch National Intensive Care Evaluation (NICE) minimal dataset, the Dutch ICU Quality Registry that is manually maintained regularly next to the hospital information system. To explore the predictor mapping discrepancy between both cohorts, we followed a staged approach for each predictor: (1) first we checked whether a predictor is part of routine care data, i.e., is it automatically processed and displayed in the EHR system; (2) then we checked if the predictor is available in the EHR system, does it need to be converted to a different unit (3), for predictors not available in the system, we checked whether the predictor components are available, and if so, (4) whether the predictor needed to be engineered or mined from the text.

**Figure 1:**
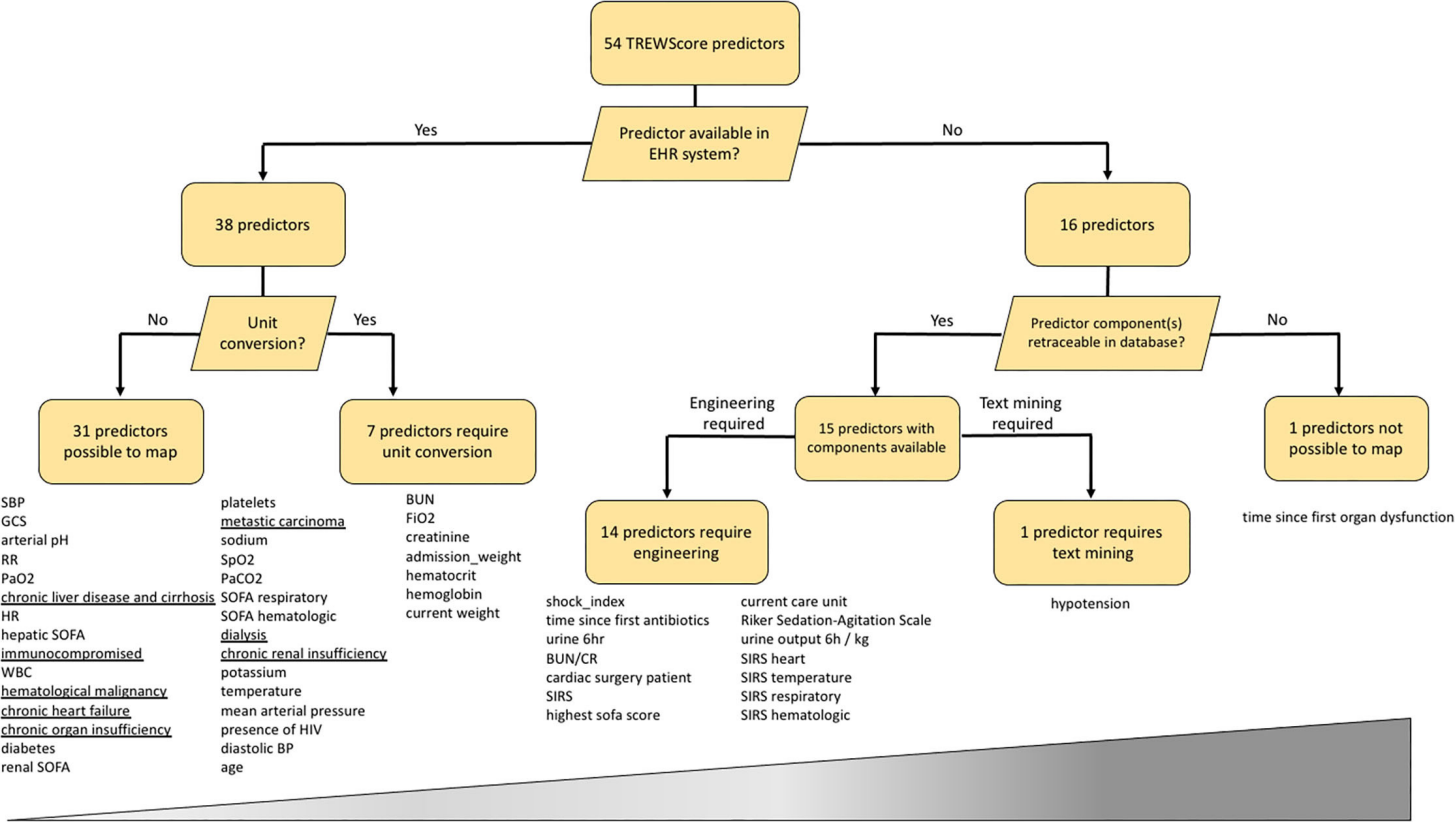
Mapping of the 54 TREWScore predictors to the UMC ICU database. Predictors are listed below each end-node. The increasing width of the bar represents the difficulty scale from easy to hard of mapping each predictor category. Underlined predictors are within 24 h documented in the UMC ICU EHR system and therefore not readily available at ICU admission. EHR, Electronic Health Record; SBP, Systolic Blood Pressure; GCS, Glasgow Coma Scale; RR, Respiratory Rate; HR, Heart Rate; SOFA, Sequential Organ Failure Assessment; WBC, White Blood Cell count; MAP, Mean Arterial Pressure; BP, Blood Pressure; BUN, Blood Urea Nitrogen; BUN/CR, BUN Creatinine Ratio; SIRS, Severe Inflammatory Response Syndrome; HIV, Human Immunodeficiency Virus.

Results on the TREWScore predictor mapping are shown in Figure 1 and Supplementary Table 1. Of the 54 predictors, 38 predictors were available in the UMC EHR system; 31 were readily available and seven required unit conversion: FiO_2_ and hematocrit from percentage to fraction, the hemoglobin from mmol/l to g/dl, admission weight and current weight from kg to pounds, blood urea nitrogen from mmol/l to mg/dl, and serum creatinine from μmol/l to mg/dl. Of the 31 readily available features, eight were based on ICD-9 codes for which we could find a surrogate in the NICE dataset. The remaining 16 predictors required other sources of information in terms of predictor engineering or text mining. The time since the first organ dysfunction (chronic or acute) predictor could not be mapped as the time of organ dysfunction was not clearly defined by Henry et al. (8). The remaining 53 predictors could be mapped by either engineering (*N* = 14) or text mining (*n* = 1). After unit conversion, all predictors were available to engineer the 14 predictors.

As real-time algorithms require a continuous feed of data to make predictions, we were interested in the data characteristics in both cohorts in terms of data availability and sampling frequency. As only a subset of the predictors was available in both cohorts, we only included the 22 numerical predictors required to apply the sepsis criteria that were available in both cohorts for this analysis. We assessed the data availability by counting the number of patients with at least one predictor measurement. Around 50% of all MIMIC-III patients had at least one measurement on a majority of the predictors (Supplementary Figure 1), whereas over 85% of the UMC ICU patients had at least one measurement available of all predictors.

The sampling frequency was assessed by calculating the average time between predictor measurements for patients with at least two measurements (Supplementary Table 2). Continuous vital predictors, i.e., DBP, FiO_2_, HR, MBP, RR, SBP, SpO_2_, and temperature were registered at a higher continuous rate in the UMC ICU than in the MIMIC-III database, whereas non-vital predictors (e.g., laboratory values) were available in similar sampling times in both cohorts (Supplementary Figure 2). Also, we computed the average of each predictor to compare differences in predictor population means between both cohorts. Sampling times were different and the population means between both cohorts. Population summary statistics of the predictors were similar in both cohorts (Supplementary Figure 3).

### Clinical Outcome Challenge

Following the methodology of Henry et al. (8), we applied the sepsis-2 criteria to identify patients progressing to the clinical outcome of shock in both cohorts (Supplementary Material Methods). From the 2,557 sepsis cases in the UMC cohort, 795 (31.3%) developed shock, whereas 3,961 (23.3%) of the 17,023 MIMIC-III sepsis cases developed shock (Table 1).

Furthermore, we computed the number of hours in ICU until shock onset, thereby evaluating the potential clinical value of implementing such a model in our center. The majority of MIMIC-III shock patients developed shock in the first 50 h of IC stay (Supplementary Figure 4). Median time to first shock event was higher for UMC ICU shock patients compared to MIMIC-III, 44.4 [IQR 21.6–136.3] vs. 19.6 [IQR 8.7–45.7] h, respectively. These findings underline differences in clinical outcomes between MIMIC-III and UMC ICU patients.

## Discussion

The transportability of algorithms built on routinely collected EHR could provide hospitals with an early warning for disease. However, here we identified three challenges relating to domain, predictors, and clinical outcome that we encountered when attempting to transport the TREWScore to our center. First, comparisons between the MIMIC-III and UMC ICU cohorts showed differences in medical severity. Second, more than two-thirds (38 out of 54) of the predictors could be readily mapped to the EHR system of our center, one quarter (15 out of 54) of the predictors would have to be compiled with either engineering or text mining and we found differences in data collection and predictor statistics between both centers. Third, the incidence of sepsis and the time to shock were different between both cohorts. These major challenges in all three domains provide evidence that the TREWScore cannot be easily transported to other centers and question the transportability of EWS in general.

Comparisons between the MIMIC-III and UMC ICU cohorts showed that UMC ICU patients are more intensively monitored in terms of measurement frequency and data availability. This illustrates a difference in clinical practice between a Dutch and a US American center as the ICU in the Netherlands is reserved for critically ill patients in a more acute state, while the American hospitals have a higher proportion of ICU beds in comparison to other countries (12). Nonetheless, sepsis prevalence in the MIMIC-III cohort was significantly higher than in the UMC ICU. Patients in the UMC ICU were evaluated daily clinically for having sepsis as part of the NICE minimal dataset in the Netherlands, whereas MIMIC-III patients were identified as having sepsis based on suspicion of infection defined by ICD-9 codes. As ICD-9 codes may suffer from administrative errors and retrospective allocation, this may have resulted in an overestimation of the sepsis incidence in MIMIC-III.

Because of the identified transportability challenges in this case study, we decided neither to try and validate TREWScore in our center nor further prepare it for implementation. First, TREWScore would require us to alter our clinical workflow to, for example, engineer and collect predictors that are not part of our routine clinical practice, which we would also need to obtain over a longer period to facilitate calibration of the model on existing data from our center. For example, comorbidities are available in the NICE dataset and can be used as surrogates for the ICD-9 codes. However, the physician is requested to document these comorbidities within 24 h after ICU admission, making these data not available at admission. In comparison to the drawbacks of ICD-9 documentation as mentioned above, this system would provide more accurate data for the score. Moreover, we found differences in data collection in terms of frequency of measurements. Lastly, it remains unclear to us how a range of laboratory variables was exactly measured. For example, whether bilirubin was measured conjugated or unconjugated, on what analyzer from what manufacturer using what assay type. Because of all limitations described in our article, we question whether the limited value for our center would outweigh our efforts to implement such a system.

Interestingly, the septic shock time to event was higher for the UMC ICU cohort in comparison with the MIMIC-III cohort. This effect can partly be explained by the longer length of stay of UMC ICU patients as compared to the MIMIC-III cohort. Moreover, the different subgroups of patients in both ICU cohorts could also explain this effect, as explained above. Furthermore, we have not stratified community vs. nosocomial onset and were not able to compare care severity between cohorts as these data were not available. These differences should be carefully explored before implementing EWS scores in clinical practice.

Data-driven ICU models have the potential to improve patient care in other centers, beyond yielding interesting research papers for literature. Here, we showed compelling evidence that there are many differences between centers in terms of domain, predictors, and clinical outcome. Our perspective resonates with recently reported challenges during external validation efforts of the Epic Sepsis Model for sepsis (13). Moreover, the lack of methodological details to reproduce research and differences in clinical practice currently further complicates transportability.

Data scientists in healthcare that make a commitment to improving patient care have the obligation to sufficiently address transportability issues. First, this means that code and data are made available in an understandable way and that description of methodology facilitates reproducibility, e.g., using current reporting guidelines (14, 15). Second, to improve the predictor mapping, researchers should adhere to data standards, such as FHIR and SNOMED CT, which should be facilitated by IT infrastructures accordingly (16, 17). Third, to further promote uptake around the globe, researchers should use predictors that are ubiquitously available in the medical domain when making models to reduce center and/or region-specific bias. These efforts should help to ameliorate the transportability of models to other centers for external validation and assessment of clinical relevance (18). Here, we show that all three transportability challenges, regarding domain, predictors, and clinical outcome, should be addressed before an EWS can be transported and used in another center. Only then, the true potential of the universal implementation of machine learning models in the intensive care can be assessed and potentially achieved for the benefit of our patients.

## Data Availability Statement

Data from the MIMIC-III version 1.4 database is publicly available on (https://mimic.physionet.org). Data from the UMC ICU is available upon reasonable request.

## Ethics Statement

The studies involving human participants were reviewed and approved by Institutional Review Board of the Utrecht Medical University Center. Written informed consent for participation was not required for this study in accordance with the national legislation and the institutional requirements.

## Author Contributions

WS, OC, and SH conceived the idea of the study and designed the study. MRJV and JV-W acquired the data for the study. MN analyzed the data, together with IH, DB, MHV, and SH. MN and SH wrote the manuscript. All authors critically reviewed the manuscript.

## Funding

MN, DB, and MHV are employees of SkylineDx. SH was funded by a fellowship of Abbott Diagnostics.

## Conflict of Interest

MN was employed by SkylineDx, Rotterdam and received a PhD fellowship from SkylineDx, Rotterdam. DB was employed by SkylineDx, Rotterdam. MVi was employed by SkylineDx, Rotterdam. SH received a fellowship from Abbott Diagnostics. The remaining authors declare that the research was conducted in the absence of any commercial or financial relationships that could be construed as a potential conflict of interest.

## Publisher's Note

All claims expressed in this article are solely those of the authors and do not necessarily represent those of their affiliated organizations, or those of the publisher, the editors and the reviewers. Any product that may be evaluated in this article, or claim that may be made by its manufacturer, is not guaranteed or endorsed by the publisher.
