# Supplementary material for: Transportability and Implementation Challenges of Early Warning Scores for Septic Shock in the ICU: A Perspective on the TREWScore
Source: Front Med (Lausanne). 2022 Feb 8;8:793815. doi: 10.3389/fmed.2021.793815 (PMC8860834; doi:10.3389/fmed.2021.793815)
Supplement: Supplementary file 1 [file Data_Sheet_1.docx]

### Supplementary Figures and Tables

### Supplementary Figures


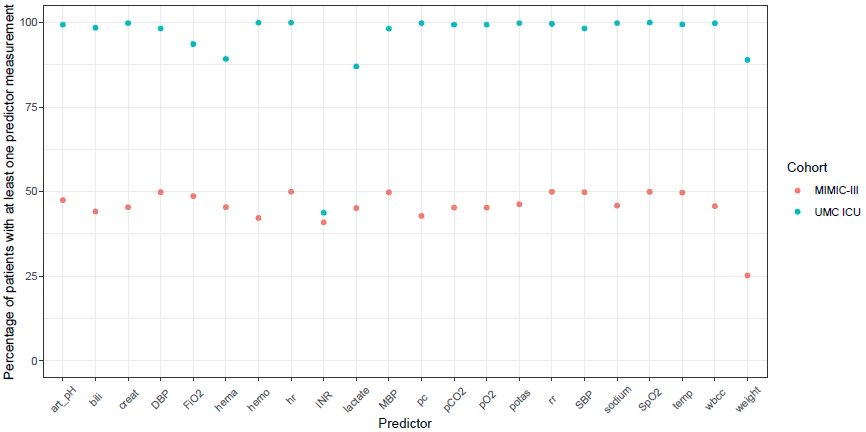


**Supplemental Figure 1: Percentage measured of all patients with at least one measurement for the 22 continuous predictors required to apply the sepsis-2 criteria definition.**


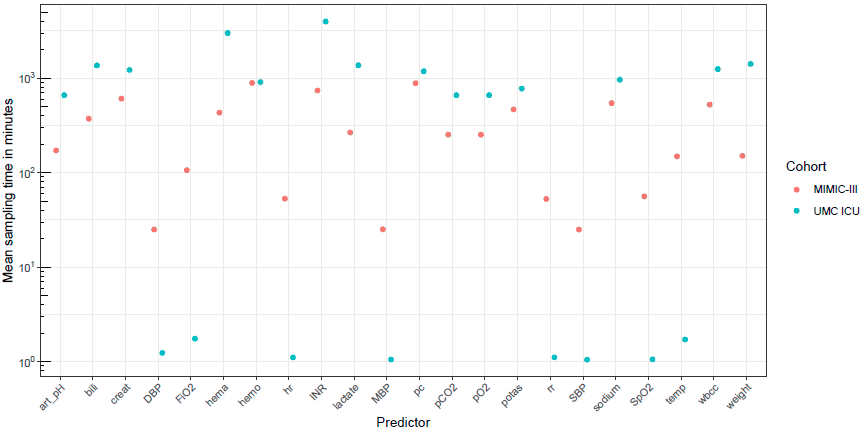


**Supplemental Figure 2: Mean sampling times in minutes for each predictor required to apply the sepsis-2 criteria definition in both cohorts expressed as relative log.**


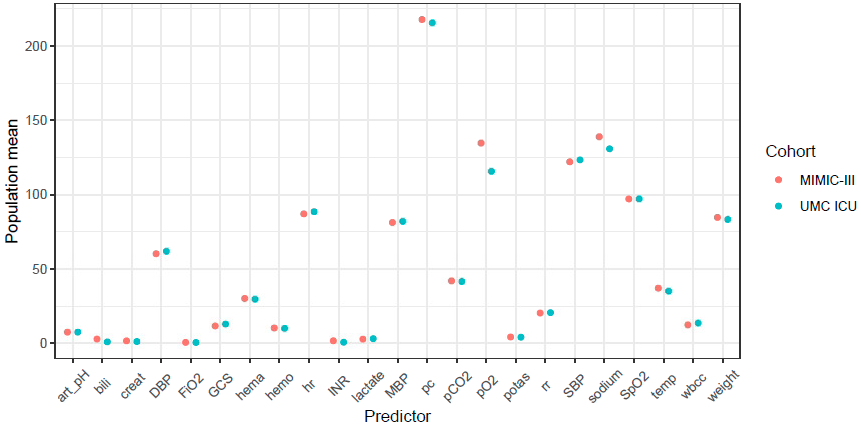


**Supplemental Figure 3: Difference in population mean for the 22 predictors required to apply the sepsis-2 criteria definition between the MIMIC-III and UMC ICU cohorts.**


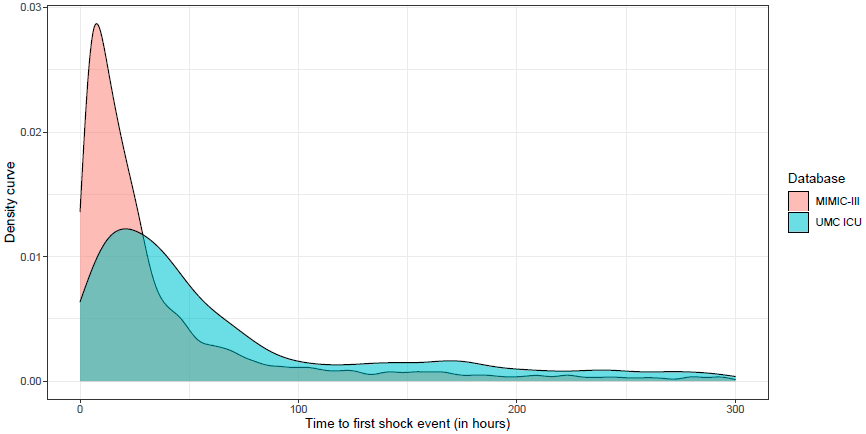


### Supplemental Figure 4: Time to first shock event for all sepsis-2 criteria identified shock cases in the MIMIC-III and UMC ICU cohorts. For aesthetic purposes, 221 observations in the right tail of the distribution are not shown.

### Supplementary Tables

### Supplemental Table 1: Mapping of the 54 TREWScore. First 26 predictors in bold are part of the TREWScore algorithm.

| Predictor | Predictor available in EHR system (unit conversion) | Predictor components readily available in UMC ICU database, required action |
| --- | --- | --- |
| **shock_index** | no | yes, computation |
| **SBP** | yes |  |
| **time since first antibiotics** | no | yes, computation |
| **urine 6hr** | no | yes, computation |
| **BUN/CR** | no | yes, computation |
| **GCS** | yes |  |
| **arterial pH** | yes |  |
| **RR** | yes |  |
| **PaO2** | yes |  |
| **BUN** | yes, (mmol/L to mg/dL) |  |
| **chronic liver disease and cirrhosis*** | yes | within 24 hours available |
| **HR** | yes |  |
| **hepatic SOFA** | yes |  |
| **FiO2** | yes (percentage to fraction) |  |
| **cardiac surgery patient** | yes | yes, computation |
| **immunocompromised*** | yes | within 24 hours available |
| **SIRS** | no | yes, computation |
| **WBC** | yes |  |
| **hematological malignancy*** | yes | within 24 hours available |
| **chronic heart failure*** | yes | within 24 hours available |
| **chronic organ insufficiency*** | yes | within 24 hours available |
| **diabetes*** | yes | available at ICU admission |
| **renal SOFA** | yes |  |
| **neurologic SOFA** | yes |  |
| **platelets** | yes |  |
| **metastatic carcinoma*** | yes | within 24 hours available |
| highest sofa score | no | yes, computation |
| sodium | yes |  |
| SpO2 | yes |  |
| PaCO2 | yes |  |
| SOFA respiratory | yes |  |
| SOFA hematologic | yes |  |
| dialysis* | yes | within 24 hours available |
| presence of chronic renal insufficiency* | yes | within 24 hours available |
| creatinine | yes (μmol/L to mg/dL) |  |
| potassium | yes |  |
| admission weight | yes (pounds to kg) |  |
| current care unit | yes | yes, computation |
| hematocrit | yes (percentage to fraction) |  |
| hemoglobin | yes (mmol/L to grams/dL) |  |
| hypotension* | no | yes, text mining |
| temperature | yes |  |
| mean arterial pressure | yes |  |
| current weight | yes (pounds to kg) |  |
| Riker Sedation-Agitation Scale | yes | yes, computation |
| presence of HIV* | yes |  |
| diastolic BP | yes |  |
| urine output the past 6 hours per kg | no | yes, computation |
| SIRS heart | no | yes, computation |
| SIRS temperature | no | yes, computation |
| SIRS respiratory | no | yes, computation |
| SIRS hematologic | no | yes, computation |
| time since first organ dysfunction | no | no |
| age | yes |  |

* TREWScore predictors based on ICD-9 code(s).

### Supplemental Table 2: Predictors required to apply the sepsis-2 criteria. Physiologically unrealistic outliers under the lower limit or above the upper limit were removed for each continuous predictor. Population predictor means computed with all ICU stays are shown for both cohorts.

| Predictor | Abbreviation | Unit | Lower limit | Upper limit | Predictor population mean MIMIC-III | Predictor population mean UMC ICU |
| --- | --- | --- | --- | --- | --- | --- |
| Arterial pH | art_ph | pH | 6 | 8 | 7.4 | 7.4 |
| Bilirubin (total) | bili | mg/dL | 0 | 20 | 2.8 | 0.9 |
| Serum creatinine | creat | mg/dL | 0 | 50 | 1.5 | 1.1 |
| Diastolic blood pressure | DBP | mmHg | 20 | 400 | 60.2 | 61.9 |
| Fraction of inspired oxygen | FiO2 | fraction | 0 | 1 | 0.5 | 0.4 |
| Glasgow Coma Scale | GCS | points | 0 | 15 | 11.6 | 12.8 |
| Hematocrit | hema | % | 0 | 100 | 30.1 | 29.6 |
| Hemoglobin | hemo | g/dL | 0 | 50 | 10.2 | 9.9 |
| Heart rate | hr | beats per minute | 5 | 350 | 87.0 | 88.5 |
| International normalized ratio | INR | ratio | 0 | 20 | 1.6 | 0.6 |
| Blood lactate | lactate | mmol/L | 0 | 50 | 2.7 | 3.0 |
| Mean blood pressure | MBP | mmHg | 20 | 400 | 81.2 | 82.0 |
| Partial carbon dioxide pressure | pCO2 | mmHg | 0 | 200 | 41.91 | 41.53 |
| Partial oxygen pressure | pO2 | mmHg | 0 | 500 | 134.7 | 115.7 |
| Platelet count | pc | K/uL | 0 | 2000 | 217.9 | 215.7 |
| Potassium | potas | mmol/L | 0 | 20 | 4.1 | 4.0 |
| Respiratory rate | rr | Beats per minute | 1 | 150 | 20.3 | 20.5 |
| Sodium | sodium | mmol/L | 0 | 1000 | 139.0 | 130.9 |
| Systolic blood pressure | SBP | mmHg | 2 | 400 | 122.1 | 123.4 |
| Oxygen saturation | SpO2 | % | 0 | 100 | 97.1 | 97.1 |
| Temperature | temp | Degrees Celsius | 10 | 50 | 37.0 | 36.9 |
| White blood cell count | wbcc | K/uL | 0.1 | 100 | 12.3 | 13.5 |
| Weight | weight | Kg | 10 | 700 | 84.7 | 83.3 |
