# Supplementary material for: Transportability and Implementation Challenges of Early Warning Scores for Septic Shock in the ICU: A Perspective on the TREWScore
Source: Front Med (Lausanne). 2022 Feb 8;8:793815. doi: 10.3389/fmed.2021.793815 (PMC8860834; doi:10.3389/fmed.2021.793815)
Supplement: Supplementary file 2 [file Data_Sheet_2.docx]

## Supplementary Material

## *Supplemental methods*

### MIMIC-III data preprocessing

MIMIC-III was downloaded and imported as PostgreSQL database. Time stamped measurement values were extracted with each predictor’s collection of item IDs into R environment using the *RpostGreSQL* R package (Supplementary Materials Table 1)(17). Predictor were extracted from the related database table as listed in Excel file. As predictors can have multiple measurements for multiple items IDs, measurements at similar time stamps were averaged for each predictor. Physiological and lab measurements were extracted for from the *chartevents* table.

As MIMIC database changed from the CareVue to the MetaVision EHR system some data had to be wrangled. Depending on the administration of data in each EHR system, urine output and fluid input had to extracted for each patient depending on EHR system. CareVue item ID 198 provides the summed GCS, whereas the MetaVision GCS item IDs 220739, 223900, 2239010 had to be summed. FiO2 item ID 223835 was converted from percentage to fraction and temperature item IDS (678, 679, 223671) were converted from Fahrenheit to Celsius degrees. In addition, units of the following predictors were converted, hematocrit from percentage to fraction, admission weight and current weight from pounds to kg, serum creatinine and bilirubin from μmol/L to mg/dl, and hemoglobin from mmol/L to grams/dL.

Determining sepsis, severe sepsis and septic shock

Sepsis-2 criteria were used to determine the sepsis stages for each ICU stay at each timepoint: non-sepsis, SIRS, sepsis, severe sepsis and septic shock.

SIRS was defined as having at least two of the SIRS criteria: temperature <36 °C or >38 °C, heart rate > 90 BPM, respiratory rate: >20 BPM or arterial CO2 pressure (PaCO2) < 32 mmHg and white blood cell count: <4,000/mL or >12,000/mL.

Sepsis was defined as having SIRS and a suspected infection defined by registration of any ICD-9 code described by Angus et al. (2001) As ICD-9 coding is not part of routine care in the UMC ICU we could not identify sepsis patients using ICD-9 codes(18). Suspicion of chronic dialysis, renal insufficiency and pneumonia comorbidities are part of the severe sepsis criteria and are also defined by ICD-9 codes. Due to the unavailability of ICD-9 codes in the UMC ICU, we used sepsis and comorbidities recorded as part of the NICE minimal dataset in the Netherlands as surrogates.

Severe sepsis was defined as having sepsis and at least one of the following criteria: SBP < 90 mm Hg, lactate > 2.0 mmol/L, < 0.5 mL/kg urine output over the past two hours despite adequate fluid resuscitation, creatinine >2.0 mg/dL without the presence of chronic dialysis or renal insufficiency as indicated by ICD-9 codes V45.11 or 585.9, bilirubin > 2.0 mg/dL, platelet count <100,000/μL, international normalized ratio (INR) > 1.5, pneumonia defined as acute lung injury with arterial O2 pressure (PaO2)/fraction of inspired oxygen (FiO2) < 200 in the presence of pneumonia as indicated by an ICD-9 code of 486, or acute lung injury with PaO2/FiO2 < 250 in the absence of pneumonia. Adequate fluid resuscitation was defined as fluid replacement over the past 24 hours >= 20 mL divided by body weights or a total fluid replacement over the previous 24 hours >= 1,200 mL.

Septic shock was defined as having severe sepsis and hypotension for more than 30 minutes with adequate resuscitation. We defined time to event as the time between the first measurement during ICU stay to the first time a patient met the criteria for septic shock.

### Predictor engineering

At ICU admission monitoring of blood pressure (BP) is initiated with an oscillometric arm cuff (non-invasive). When a patient’s conditions worsen an arterial catheter is inserted (invasive) as this technique is more accurate and reliable. Measurements from both methods for all three blood pressure types (systolic, mean, diastolic) were available in both databases. As BP monitoring measurement types could overlap specific time points, both non-invasive and invasive measurements were combined with the focus on the invasive monitoring when measurements from both methods were available. For both cohorts, invasive blood monitoring was determined when the patient had at least one invasive blood monitoring measurement regardless of type. Likewise, mechanical ventilation was determined when at least one record of mechanical ventilation during stay was administrated during ICU stay.

### Imputation

As not all predictors required to apply the sepsis-2 criteria were continuously measured in both centers, a lot of predictors did not have measurements for each timestamp. After removal of the physiologically unrealistic outliers (Supplemental Table 1), we imputed missing values by first defining three types of missing values: (1) in-between and (2) after missing values, as well as (3) predictors that were not measured for a patient. Values that were missing in-between observations were imputed with a carry forward imputation technique. Multiple techniques are used in literature for the other two types of missing values(19,20). In line with Henry et al. (2015), we imputed predictors missing completely with the population mean. As Henry et al. (2015) did not specify how they imputed the before values, we used the predictor’s population mean to impute these missing values. Predictor population means were computed for each cohort by using all predictor measurements from the complete cohort.

### ICD-9 predictors

ICD-9 predictors (N = 9)

- Presence of chronic liver disease and cirrhosis as indicated by ICD-9 571
- immunocompromised (patient has received past therapy that suppresses resistance to infection) as indicated by presence of any ICD-9 in V58.65, V58.0, V58.1, 042, 208.0, 202
- presence of hematologic malignancy as indicated by any ICD-9 code in 200-208
- presence of heart failure as indicated by ICD-9 code 428
- severe organ insufficiency (chronic liver disease, chronic heart failure, chronic respiratory failure, receiving chronic dialysis) as indicated by one of the ICD-9 codes 571, 585.6, 428.22, 428.32, 428.42, 518.83
- presence of diabetes as indicated by ICD-9 code 250
- metastatic carcinoma as indicated by presence of any ICD-9 codes in 140-165, 170-165, 179-199
- Dialysis (not defined by Henry et al. (2015))
- Presence of chronic renal insufficiency (not defined by Henry et al. (2015))
- presence of HIV (not defined by Henry et al. (2015))
- Hypotension (unclear whether ICD-9 code or sbp < 90 mm Hg)

### Analysis

All analyses were performed using R version 3.6.1.
